# Zoledronate Effects on Systemic and Jaw Osteopenias in Ovariectomized Periostin-Deficient Mice

**DOI:** 10.1371/journal.pone.0058726

**Published:** 2013-03-07

**Authors:** Nicolas Bonnet, Philippe Lesclous, Jean Louis Saffar, Serge Ferrari

**Affiliations:** 1 Division of Bone Diseases, Department of Internal Medicine Specialties, Geneva University Hospital, Geneva, Switzerland; 2 Laboratoire Pathologies, Imagerie et Biothérapie de l′organe dentaire EA 2496, Université Paris-Descartes, Faculté de Chirurgie Dentaire, Paris, France; 3 INSERM-UMRS 791, LIOAD: Laboratoire d′Ingénierie Ostéo-Articulaire et Dentaire, STEP: Skeletal Tissue Engineering and Physiopathology group, Nantes University, School of Dentistry, Hôtel Dieu Hospital, LUNAM Université, Nantes, France; Georgia Health Sciences University, United States of America

## Abstract

Osteoporosis and periodontal disease (PD) are frequently associated in the elderly, both concurring to the loss of jaw alveolar bone and finally of teeth. Bisphosphonates improve alveolar bone loss but have also been associated with osteonecrosis of the jaw (ONJ), particularly using oncological doses of zoledronate. The effects and therapeutic margin of zoledronate on jaw bone therefore remain uncertain. We reappraised the efficacy and safety of Zoledronate (Zol) in ovariectomized (OVX) periostin (Postn)-deficient mice, a unique genetic model of systemic and jaw osteopenia. Compared to vehicle, Zol 1M (100 µg/kg/month) and Zol 1W (100 µg/kg/week) for 3 months both significantly improved femur BMD, trabecular bone volume on tissue volume (BV/TV) and cortical bone volume in both OVX Postn^+/+^ and Postn^−/−^ (all p<0.01). Zol 1M and Zol 1W also improved jaw alveolar and basal BV/TV, although the highest dose (Zol 1W) was less efficient, particularly in Postn^−/−^. Zol decreased osteoclast number and bone formation indices, i.e. MAR, MPm/BPm and BFR, independently in Postn^−/−^ and Postn^+/+^, both in the long bones and in deep jaw alveolar bone, without differences between Zol doses. Zol 1M and Zol 1W did not reactivate inflammation nor increase fibrous tissue in the bone marrow of the jaw, whereas the distance between the root and the enamel of the incisor (DRI) remained high in Postn^−/−^ vs Postn^+/+^ confirming latent inflammation and lack of crestal alveolar bone. Zol 1W and Zol 1M decreased osteocyte numbers in Postn^−/−^ and Postn^+/+^ mandible, and Zol 1W increased the number of empty lacunae in Postn^−/−^, however no areas of necrotic bone were observed. These results demonstrate that zoledronate improves jaw osteopenia and suggest that in Postn^−/−^ mice, zoledronate is not sufficient to induce bone necrosis.

## Introduction

Periodontal disease (PD) is a common problem as it is estimated that 48.2% of the US population aged more than 30 years is affected, with up to 20% of subjects showing severe tooth attachment loss in the NHANES III survey [1]. Moreover, PD seems to be frequently associated with osteoporosis [2], which could further aggravates the risk of tooth loosening [3], [4]. Periodontal disease, especially measured by alveolar bone loss, is a strong predictor for incident of tooth loss in postmenopausal women, approximately one-third of adults age 65 and older losses one or more tooth [5]. Bisphosphonates, particularly alendronate and risedronate, have been shown to exert favorable effects on PD [6], [7]. Clinical studies demonstrated a reduction of radiological and/or clinical signs of PD, including alveolar bone loss, gingival index and bleeding [8], [9], [10], [11], [12]. These beneficial effect of bisphosphonates on mandibular bone loss have also been demonstrated in animal models of PD such as the rice rat [13], [14], and/or as induced by elastic rings [15], [16], tooth ligature [7], [17], and bacterial inoculations [18], [19].

On another side, the occurrence of osteonecrosis of the jaw (ONJ) in some patients receiving bisphosphonates, particularly high-dose zoledronate for the prevention of skeletal complications of malignancy, has raised important questions regarding the role of these drugs in the development of ONJ [20], [21]. Tooth extraction is commonly considered as a precipitating factor for bisphosphonates-related osteonecrosis of the jaw (BRONJ) [22], [23]. However apparently spontaneous BRONJ, i.e. without any surgical traumatism of alveolar bone, also occurs. Nethertheless, it seems that PD is a crucial factor in the development of ONJ in patients receiving bisphosphonates. Indeed, oral preventive measures avoiding tooth mobility, periodontal diseases, presence of root fragments, decays, granulomas, edentulism and periapical conditions [24] decrease ONJ incidence [25], [26]. Consistent with these clinical observations, lesions reminiscent of ONJ have been observed following administration of high-dose zoledronate in rats with severe PD [27], [28]. Hence tooth ligature and cumulative high doses of zoledronate in rats (66 µg/kg, i.e equivalent to 4 mg/60 kg in humans, three times per week for 3 weeks) led to the appearance of necrotic bone in the mandible [27], arguing that both the presence of infectious/inflammatory sockets and lack of bone remodeling due to bisphosphonates concur to the development of ONJ-like lesions, despite a significant reduction of alveolar bone loss [27]. Similarly, in the rice rat model (Oryzomys Palustris) with periodontitis, high dose zoledronate (80 µg/kg I.V, once monthly during 18 and 24 weeks) induced ONJ-like lesions characterized by areas of exposed necrotic alveolar bone, increased osteocyte apoptosis and presence of bacterial colonies [28]. Whether the start of the necrosis process is secondary to PD [27] or is primarily an aseptic process [29], however remains questionable.

Several drug-related mechanisms have been suggested to eventually explain the contribution of bisphosphonates to the development of ONJ, all so far unproven, including an impairment of soft tissue and/or bone vascularization; a direct toxicity on the gingival epithelium; a direct toxicity on bone cells, particularly osteocytes; and of course the suppression of bone turnover [30]. Experimental models of aggressive PD (above) however do not allow to clarify the potential pathophysiological role of bisphosphonates in the development of ONJ beyond the absence of removal of necrotic bone fragments induced inflammation/infection. Whether bisphosphonates can actually cause jaw bone necrosis in absence of injury/inflammation, as observed in some patients without tooth extraction or severe PD, therefore remains debatable.

Periostin deficient mice (*Postn^−/−^*) represent a unique opportunity to reappraise the effects of bisphosphonates on the jaw. Periostin is a matricellular protein synthesized by osteoblast/osteocytes, that is linked to type I collagen in the periodontal ligament, where it regulates fibrillogenesis [31], [32]. Periostin-deficient mice show severe alterations in tooth eruption, resulting from a failure to digest collagen fibers in the shear zone of the periodontal ligament [33]. As a consequence, the enamel and dentin of the incisors are compressed and disorganized, and these mice develop inflammatory infiltrates (particularly neutrophils) in the periodontal ligament, loss of crestal (superficial) alveolar bone and alveolar bone loss underneath. The severity of PD can be moderated by feeding the mice a soft diet, indicating that the trigger of the inflammatory infiltrate in the periodontal ligament is traumatic mastication [33]. On a soft diet, *Postn^−/−^* mice do not have overt PD with inflammation, however they still present alveolar and systemic osteopenia [34]. Accordingly, we used old *Postn^−/−^* mice kept on a soft diet to reappraise the intrinsic effects of high dose zoledronate on the jaw. Here we hypothesize that zoledronate will improve jaw and systemic osteopenia, without causing necrosis of alveolar bone in absence of overt PD. Our results indicate that zoledronate improves jaw and systemic osteopenia without causing alveolar bone necrosis in absence of overt PD, despite a decrease in osteocyte number.

## Materials and Methods

### Animals

Postn Lac Z knock-in mice (*Postn^−/−^*) were generated as reported previously [33]. *Postn^−/−^* mice were subsequently bred with C57BL/6J mice, and DNA analyzed by PCR was used to identify Postn heterozygous mice. We interbred mice that were heterozygous carriers of this mutation and obtained wild-type (*Postn^+/+^*) and homozygous mutant (*Postn^−/−^*) offspring with the expected Mendelian distribution. They were subsequently back-crossed for 10 generations, resulting in a genome of 99% C57BL/6J. Mice were housed five per cage, maintained under standard non barriers conditions and had access to water and a soft diet ad libitum (Harlan Teklad 2019,SDS, England). Under those experimental conditions, the malnutrition that is otherwise observed in the *Postn^−/−^* mice under a standard (hard) diet that causes the enamel and dentin defects of the incisors and molars [33], is partially prevented. This dietary adaptation also reduces the severity of PD [33], i.e. inflammation. All mice received the same diet during the experiment. Ten month-old female *Postn^−/−^* and *Postn^+/+^* mice were OVX (8/gr) or Sham-operated (6/gr) and administered directly after the surgery subcutaneous Zol either once weekly (Zol 1W, 100 µg/kg/week), or once monthly (Zol 1M, 100 µg/kg/month), or vehicle (Veh) for 3 months, corresponding to a cumulative dose of 16 mg/60 kg/month (ZOL 1W) or 4 mg/60 kg/month (ZOL 1M), respectively. These drug exposures correspond to 4 times, and 1 time respectively, of the monthly dose of zoledronate prescribed for the prevention of skeletal-related events in cancer patients [27], [35]. The duration of treatment has been chosen based on previous study describing the structural effects of Zol in rodents [36]–[38]. Lumbar spine, femurs and mandible were excised and stored in ethanol for micro-computed tomography analysis & histomorphometry and kept at −20°C for biomechanical analysis. All surgical procedures were performed under ketamin/xylasin, and OVX animals received Buprenorphin (0.05 mg/kg) just after surgery and every 12 h for 2days. Ethics Statement: animal procedures were approved by the University Of Geneva School Of Medecine Ethical Committee and the State of Geneva Veterinarian Office.

### *In vivo* measurement of bone mineral density

Total body, femoral and spinal bone mineral density (BMD, g/cm^2^) were measured *in vivo* at 10, 11.5 and 13 months of age by dual-energy X-ray absorptiometry (PIXImus2, GE lunar, Madison WI) [39].

### *Ex vivo* measurement of morphology and microarchitecture

Micro-computed tomography (microCT UCT40, Scanco Medical AG, Basserdorf Switzerland) was used to assess trabecular bone volume fraction and microarchitecture in the excised 5^th^ lumbar spine body and distal femur, and cortical bone geometry at the midshaft femoral diaphysis as previously described [40]. Briefly, trabecular and cortical bone regions were evaluated using isotropic 12 µm voxels.

For the vertebral trabecular region, we evaluated 250 transverse CT slices between the cranial and caudal end plates, excluding 100 µm near each endplate. For the femoral and tibial trabecular region, to eliminate the primary spongiosa, we analyzed one hundred slices from the 50 slices under the distal growth plate.

Femoral cortical geometry was assessed using 50 continuous CT slides (600 µm) located at the femoral midshaft. Images were segmented using an adaptative-iterative thresholding approach rather than a fixed threshold. Morphometric variables were computed from binarized images using direct, three-dimensional techniques that do not rely on prior assumptions about the underlying structure [41]. For the trabecular bone regions, we assessed the bone volume fraction (BV/TV, %), trabecular thickness (TbTh, µm), trabecular number (TbN, mm^−1^), trabecular connectivity density (Tb Conn Density, mm^−3^) and structural model index (SMI). The structure model index was measured to determine the prevalence of plate-like or rod-like trabecular structures, where 0 represents ‘plates’ and 3 ‘rods’ [41]. For cortical bone at the femoral and tibial midshaft, we measured the cortical tissue volume (CtTV, mm^3^), bone volume (CtBV, mm^3^), the marrow volume (BMaV, mm^3^) and the average cortical width (CtTh, µm).

**Figure 1:**
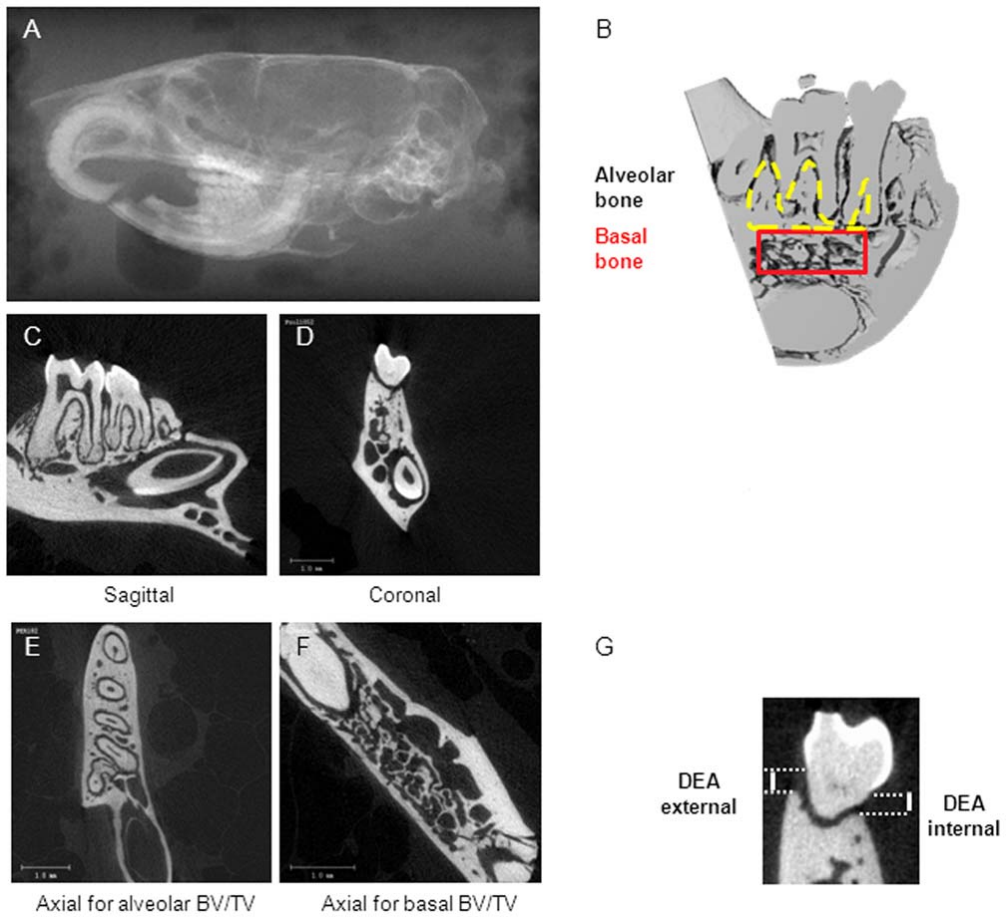
2D and 3D images of microcomputed tomography. (A) Scout view of the *Postn^−/−^* mice showing alteration of the enamel (*) that affects the incisor from the apex to the incisor edge. (B) 3D reconstruction of our region of interest for the alveolar and basal bone compartment. (C) Sagittal slice (D) coronal slice (E) Axial slice at the alveolar bone compartment (F) Axial slice at the basal bone compartment. (G) Illustration of the external and internal Distance between cement-Enamel junction and Alveolar bone (DEA), an index of exposed bone in the oral cavity.

For mandible alveolar and basal bone volume fraction (BV/TV) analysis, the head of the mice was oriented with the nasal cavity floor parallel to the horizontal plane and the midpalatal suture parallel to the midsagital plane to obtain axial CT slides (Fig. 1).

The Distance between cementum-Enamel junction and Alveolar bone (DEA), an index of alveolar bone loss, was measured at the mesial root of M1 on coronal CT slides, which represents the mean of external and internal DEA (Fig. 1).

### Histomorphometry

To measure dynamic indices of bone formation, mice received subcutaneous injections of calcein (20 mg/kg, Sigma, Buchs, Switzerland) 9 and 2 days before euthanasia.

Femur were embedded in methyl-methacrylate (Merck, Darmstadt, Germany) as previously described [42] [34], and 20-µm-thick transversal sections of the midshaft were cut with a saw (FinOcut, Metkon, Instruments LTD) than sanded to 10-µm-thick and mounted unstained for evaluation of fluorescence. Five-8 µm thick sagital sections were cut with a Leica Corp. Polycut E microtome (Leica Corp. Microsystems AG, Glattburg, Switzerland) and stained with modified Goldner's trichrome, and histomorphometric measurements were performed on the secondary spongiosa of the distal femur metaphysis and on the endocortical and periosteal bone surfaces in the middle of the femur, using a Leica Corp. Q image analyser at 40× magnification. All parameters were calculated and expressed according to standard formulas and nomenclatures [43] : mineral apposition rate (MAR, µm/day), single labeled surface (sLS/BPm, %), and double-labeled surface (dLS/BPm, %). Mineralizing surface per bone surface (MS/BPm, %) was calculated by adding dLS/BPm and one-half sLS/BPm. Bone formation rate (BFR/BPm, µm^3^/µm^2^/day) was calculated as the product of MS/BPm and MAR. Three slides per sample were analyzed for the bone formation indices. Osteocyte number and lacunae per bone area were counted on two toluidine blue stained slides of the midshaft cross-sectionnal femur per sample.

The right mandible was removed and fixed in cold (4°C) 70% ethanol. After dehydratation, the bone samples were embedded without demineralization in methylmethacrylate and polymerized at – 20° for 48 h [44]. Longitudinal sections (4 µm thick) were cut with a polycut E microtome (Leica, Wetzlar, Germany). They were stained with toluidine blue (pH 3.8) or Sirius red or hematoxylin-eosin or processed for tartrate-resistant acid phosphatase (TRAP) revelation. Two sections for each stained sample were quantified per animal. Toluidine blue staining was used to visualize osteocytes and their lacunae (expressed in number per square millimeter of trabecular bone surface) in the zone of interest, i.e the trabecular bone segment comprised between the end of the M1 root and the underlying incisor. Empty osteocyte lacunae were evaluated and necrotic bone defined as a loss of more than five contiguous osteocytes with confluent areas of empty lacunae [27]. Sirius red staining, specific for tissue collagen, was used to visualize the fibrous network in the bone marrow area. Hematoxilin-eosin was used to characterize inflammatory cells and further quantify areas of osteonecrosis. Distance between the Root and the enamel of the Incisor (DRI) was also evaluated on the hematoxilin-eosin stained sections.

TRAP was detected by using hexazotized pararosanilin (Sigma, St Louis, MO) and naphtol ASTR phosphate (Sigma, St Louis, MO) to reveal osteoclasts, ; non-osteoclastic acid phosphatase was inhibited by adding 100 mMol/L L(+)-tartric acid (Sigma, St Louis, MO) to the substrate solution. The following parameters were recorded: the number of TRAP+ osteoclasts in contact with trabeculae (N.Oc/TBPm ; expressed in cells per millimeter of trabecular bone surface) ; the resorption surface (OcS/BS ; expressed in %) ; the average length of the zone of contact per osteoclast (Oc.Pm/N.Oc ; given in microns) considered as a sensitive indicator of osteoclast activity [45]. Dynamic indices of bone formation were evaluated as described above for the femur.

### Testing of mechanical resistance

The night before mechanical testing, bones were thawed slowly at 7°C and then maintained at room temperature. The length of the femur (distance from intermalleolar to intercondylar region) was measured using callipers with an integrated electronic digital display and the midpoint of the shaft was determined. The femur then was placed on the material testing machine on two supports separated by a distance of 9.9 mm and load was applied to the midpoint of the shaft, thus creating a three-point bending test. Between each preparation step, the specimens were kept immersed in physiological solution. The mechanical resistance to failure was tested using a servo-controlled electromechanical system (Instron 1114, Instron corp., High Wycombe, UK) with actuator displaced at 2 mm/minute. Both displacement and load were recorded. All tested samples (n = 8 per group) have been included in the analysis. Ultimate force (maximal load, measured in Newtons [N]), stiffness (slope of the linear part of the curve, representing the elastic deformation, N/mm), and energy (surface under the curve, N*mm) were calculated. Ultimate stress (N/mm^2^) and Young's modulus (MPa) were determined by the equations previously described by Turner and Burr [46]. Reproducibility was 3.3% for midshaft femur as previously described elsewhere [47].

### Data analysis

The power calculation to perform our study was based on our primary criteria, the improvement of bone alterations by Zol. We first tested the effects of Zol within groups (*Postn^−/−^* and *Postn^+/+^*) by unpaired t-tests. To compare the effects of genotype and the response to treatments Zol, we used a two way ANOVA. As appropriate, post hoc testing was performed using Fisher's protected Least Squares Difference (PLSD). Differences were considered significant at p<0.05. Data were presented as mean ± SEM.

## Results

### Jaw bone alterations in *Postn^−/−^* mice

**Figure 2:**
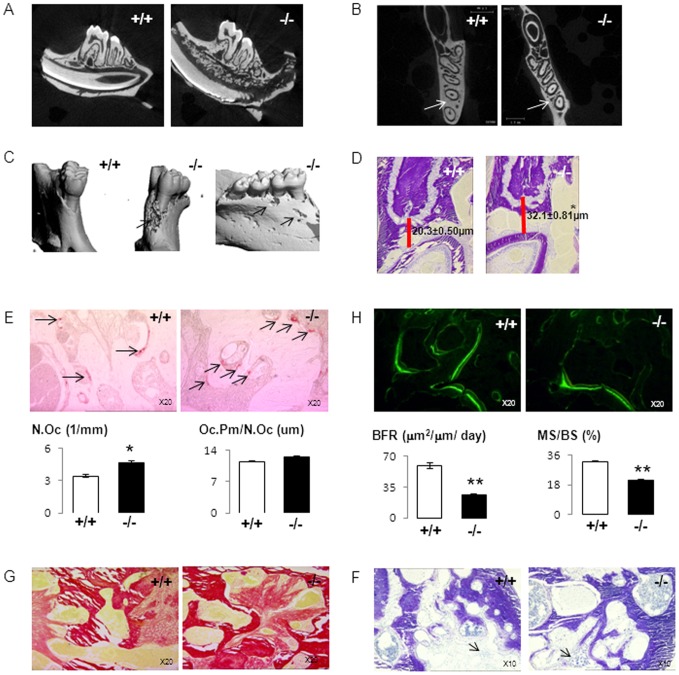
Periodontal disease disturbance and mandibule osteopenia in *Postn^−/−^* mice aged of 13 months. (A,B) Sagital and axial 2D image, arrows indicate alteration of the alveolar bone between roots in *Postn^−/−^* vs *Postn^+/+^*. (C) 3D reconstruction of the mandible, arrows shows loss of the alveolar bone and therefore tooth exhibition in *Postn^−/−^* vs *Postn^+/+^*. (D) Toluidine blue staining, red bar shows a lower distance between the root and the enamel of the incisor in *Postn^−/−^* vs *Postn^+/+^*. (E) TRAP staining, arrow indicate higher osteoclast in *Postn^−/−^* vs *Postn^+/+^* mice, Number of Osteoclast (N.Oc), activity of the osteoclast illustrated by the ratio Osteoclast Perimeter on Osteoclast Number (Oc.Pm/N.Oc), * p<0.05. (H) Fluorescent calcein labels on alveolar surfaces, Bone Formation Rate (BFR), Mineralizing Surface per Bone Surface (MS/BS). (G) Sirius red staining. (F) Toluidine blue staining, no sign of active inflammation are noted, arrows indicate residual inflammation around the periodontal incisive ligament.

*Postn^−/−^* mice fed a hard diet develop severe PD, with bone and teeth alterations [33]. To assess the severity of these alterations in *Postn^−/−^* mice receiving a soft diet, dissected mandibles of 13-months-old intact mice were imaged by histological sections and microCT. 2D and 3D microCT images illustrate alterations of the crestal alveolar bone as well as the deeper alveolar bone and cementum-enamel junction, particularly around the proximal root of the first molar (Fig. 2A–C). Quantitatively, *Postn^−/−^* present lower basal and alveolar BV/TV (−31.6% and –21.4% vs *Postn^+/+^*, p<0.01). The alteration of alveolar bone was confirmed histologically by the higher distance between the molar root and the enamel of the underlying incisor, ‘DRI’ (Fig. 2D). In this bone area, *Postn^−/−^* mice presented a higher osteoclast number (4.67±0.34, 1/mm vs 3.42±0.14, 1/mm in *Postn^+/+^* mice, p<0.01) and lower bone formation indices (Fig. 2E–H, table 1). Sirius red staining indicated large medullar spaces filled by fibrous tissue in almost all *Postn^−/−^* mice, whereas no fibrous tissue was detectable in *Postn^+/+^* mice (Fig. 2G), suggesting that an inflammatory episode had previously occurred in the mandible of *Postn^−/−^* mice. Consistent with previous descriptions [33], *Postn^−/−^* mice showed some inflammatory infiltrates around the periodontal ligament (Fig. 2F). In contrast, and likely due to the effects of a soft diet, no signs of active inflammation were noted in gingival, periradicular and jaw bone compartments at 13 months of age (Fig. 2F). These observations indicate that *Postn^−/−^* mice fed a soft diet present alveolar and systemic osteopenia without overt PD.

**Table 1:** Influence of zoledronate on bone turnover/remodelling indices in femur and jaw.

	Parameters	*SH Postn^+/+^*	Veh	*OVX Postn^+/+^* Zol1M	Zol1W	*SH Postn^−/−^*	Veh	*OVX Postn^−/−^* Zol1M	Zol1W
FEMUR	Ps MAR (µm/day)	0.49±0.04*	0.26±0.09	0.37±0.07	0.18±0.05	0.29±0.09^#^	0.23±0.11	0.17±0.05^#^	0±0^#^
*Periosteal*	Ps BFR/BPm (µm^2^/µm/ day)	0.10±0.02	0.04±0.03	0.05±0.02	0.01±.002	0.02±0.01^#^	0.04±0.03	0.01±0.004	0±0
	Ps MPm/BPm (%)	0.20±0.03^**^	0.06±0.02	0.13±0.04	0.05±0.02	0.08±0.02^#^	0.10±0.05	0.07±0.02	0.009±0.002
									
*Endocortical*	Ec MAR (µm/day)	0.77±0.05	0.63±0.07	0.02±0.02^****^	0±0^****^	0.41±0.09^#^	0.48±0.04^#^	0.29±0.04^****^	0±0^****^
	Ec BFR/BPm (µm^2^/µm/ day)	0.24±0.03	0.16±0.03	0.001±0.001^****^	0±0^****^	0.11±0.03^#^	0.15±0.03	0.002±0.002^***^	0±0^****^
	Ec MPm/BPm (%)	0.32±0.04*	0.27±0.04	0.06±0.01^****^	0.02±0.003^****^	0.22±0.04	0.30±0.04	0.10±0.01^***^	0.03±0.006 ^****^
JAW									
*Alveolar*	Tb MAR (µm/day)	1.87±0.21	1.43±0.12	1.07±0.14^****^	1.03±0.1^****^	1.27±0.06^###^	1.18±0.08^###^	0.99±0.03^***^	0.94±0.04^****^
	Tb BFR/BS (µm^2^/µm^3^/ day)	59.11±3.35	25.16±1.03	15.01±1.60^****^	14.08±1.55^****^	20.66±1.50^###^	18.33±2.23^##^	14.41±1.82^***^	13.33±1.75^***^
	Tb MS/BS (%)	32.33±2.1	36.36±2.52	16.13±0.97^**^	14.31±0.95^***^	26.29±1.48^#^	21.65±1.16^#^^#^	14.22±0.58^****^	12.43±1.63^****^

* p<0.05, ^**^p<0.01, ^***^p<0.001, ^****^p<0.0001 vs OVXvehicle of the respective genotype. ^#^p<0.05 vs *Postn^+/+^* of the respective treatments. Means ± SEM. Ps: periosteum, Ec: endocortical, Tb: trabecular. mineral apposition rate (MAR), mineralisation perimeter (MPm), mineralisation surface (MS), Bone formation rate (BFR), bone perimeter (BPm), bone surface (BS). Ovariectomitzed mice (OVX n = 8/gr) or Sham-operated (SH n = 6/gr).

### Effects of zoledronate

#### Bone structure

As previously described in male *Postn^−/−^* mice [34], both intact and OVX female *Postn^−/−^* mice had lower femur BMD, trabecular bone microarchitecture (BV/TV) and cortical bone volume (CtBV) compared to *Postn^+/+^* (−13.8%, −83.0% and −15.4% respectively, all p<0.01, Table 1). Bone formation indices were also decreased in the femur and mandible of *Postn^−/−^* mice (Table 1).

**Figure 3:**
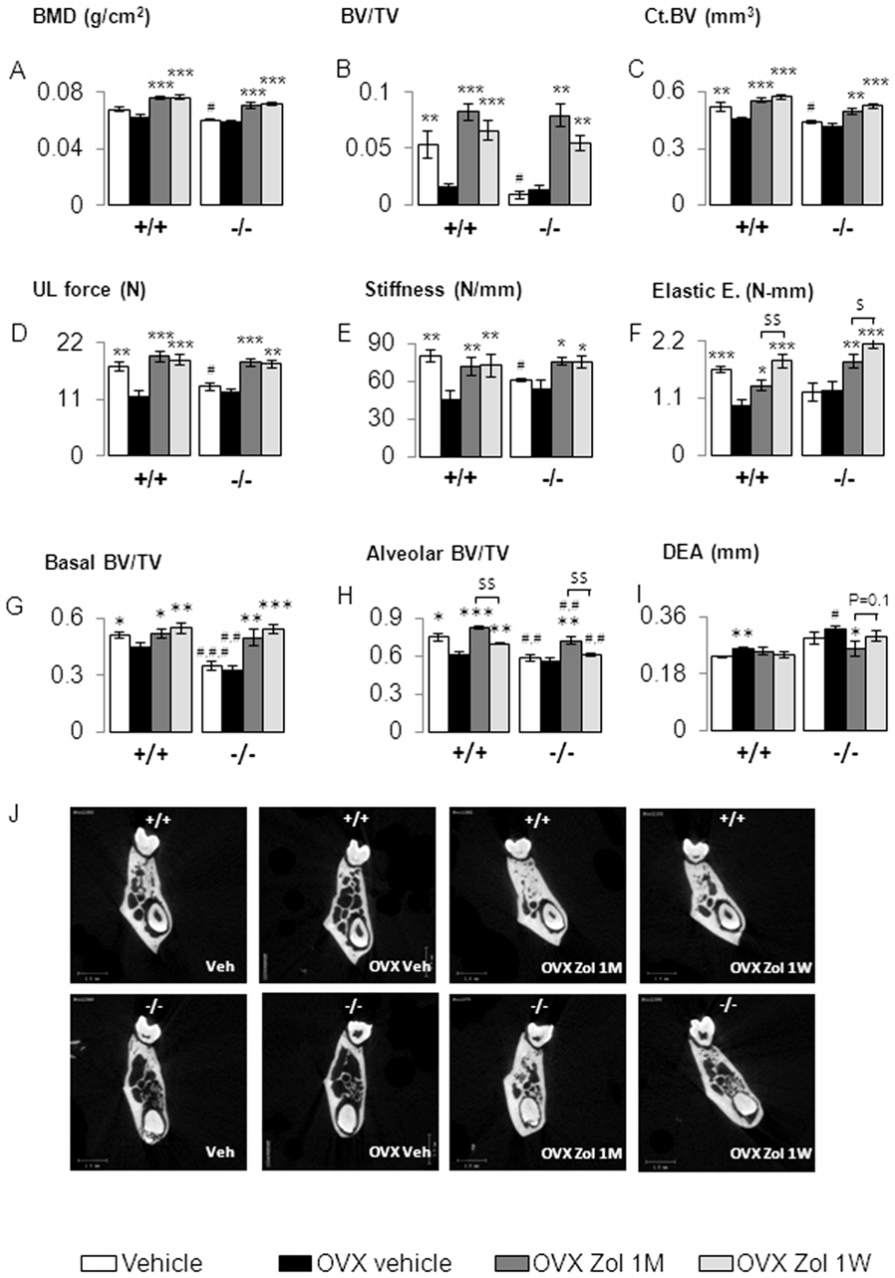
Zoledronate effect on the femur and mandible site in *Postn^+/+^* and *Postn^−/−^* mice. (A) Femur Bone Mineral Density (BMD). (B) Trabecular bone volume on tissue volume (BV/TV) of the distal femur. (C) Cortical Bone Volume (CtBV). (D, E, F) Biomechanical parameters obtain by three points bending test, (ultimate, UL; energy, E). (G, H) Basal bone volume fraction and alveolar bone volume fraction respectively obtains by microcomputed-tomography. (I) Mean of the internal and external Distance between cement-Enamel junction and Alveolar bone (DEA). (J) 2D coronal illustration of the effect of zoledronate one injection per month (Zol 1M) and one injection per week (Zol 1W) in ovariectomized (OVX) *Postn^+/+^* and *Postn^−/−^* mice. * p<0.05, ** p<0.01, *** p<0.001 vs OVX vehicle in the respective genotype of *Postn^+/+^* and *Postn^−/−^*. # p<0.05, ## p<0.01, ### p<0.001 vs *Postn^+/+^* in the respective treatment. $ p<0.05, $$ p<0.01 significant differences between OVX Zol 1M and OVX Zol 1W. Bars show means (± sem).

Compared to vehicle, Zol 1M (100 µg/kg/month) and Zol 1W (100 µg/kg/week) significantly improved BMD, femur BV/TV and CtBV in both OVX Postn^+/+^ and Postn^−/−^ mice (Fig. 3A–C, all p<0.01). Accordingly, biomechanical properties of the femur were improved by Zol in both genotypes (Fig. 3D–F).

**Figure 4:**
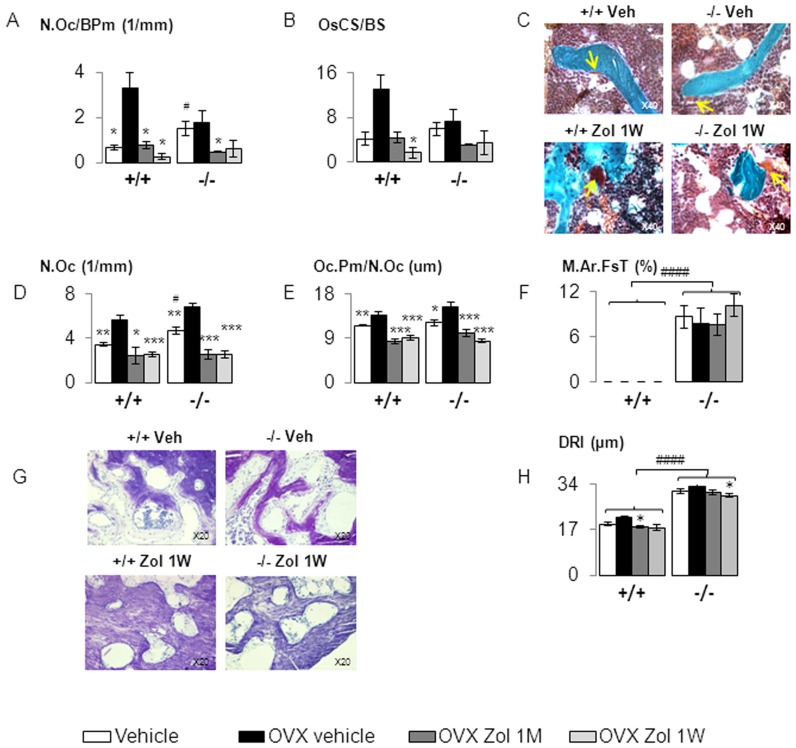
Zoledronate effect on bone resorption and sign of inflammation in *Postn^+/+^* and *Postn^−/−^* mice. (A–C) Histomorphometry parameter and images perform on the femur. (A) Number of Osteoclast on Bone Perimeter (N.Oc/BPm), (B) Osteoclast Surface on Bone Surface (OcS/BS), (C) Goldner staining, yellow arrows indicate normal size of osteoclast in vehicle groups and shows giant osteoclast under zoledronate treatment either in *Postn^+/+^* and *Postn^−/−^* mice. (D–H) Histomorphometry parameter and images perform on the mandible. (D) Osteoclast Number (N.Oc), (E) Osteoclast Perimeter on Osteoclast Number (Oc.Pm/N.Oc). (F) Medullary area filled by fibrous tissue (M.Ar.FsT). (G) Toluidine blue staining, note that Zol1W do not active inflammation. (H) Distance between the Root and the enamel of the Incisor (DRI). * p<0.05, ** p<0.01, *** p<0.001 vs OVX vehicle in the respective genotype of *Postn^+/+^* and *Postn^−/−^*. # p<0.05, ## p<0.01, #### p<0.0001 vs *Postn^+/+^* in the respective treatment. Bars show means (± sem).

In the mandible, Zol 1W and 1M also improved basal BV/TV both in Postn^−/−^ and Postn^+/+^ mice (p<0.01) (Fig. 3G). As a consequence the distance between the molar root and the enamel of the underlying incisor (DRI) decreased in response to Zol in both Postn^−/−^ and Postn^+/+^ (Fig 4H). Zol 1M also significantly increased alveolar BV/TV vs Veh in Postn^−/−^ (+27%, p<0.01) and in Postn^+/+^ (+35%, p<0.001) (Fig. 3H & 3J). However, Postn^−/−^ and Postn^+/+^ mice treated with Zol 1W had significantly lower alveolar BV/TV vs Zol 1M (all p<0.01). Moreover, Zol 1M decreased vs Veh the Distance between cement-Enamel junction and Alveolar bone (DEA) in Postn^−/−^ (p<0.05, Fig. 3I), whereas Zol 1W did not.

#### Bone remodelling

Zol decreased osteoclast number and surface in Postn^−/−^ and Postn^+/+^, both in the long bones and in deep jaw alveolar bone (Fig. 4A–E), without differences between Zol doses. As previously described, we confirmed the presence of giant osteoclasts with Zol in both Postn^−/−^ and Postn^+/+^ (Fig. 4C). Zol did not affect bone formation indices at the periosteum either in Postn^−/−^ nor Postn^+/+^ mice (Table 1). In contrast Zol at both doses significantly decreased bone formation indices, i.e. MAR, MPm/BPm and BFR, at the endocortical and alveolar compartments in Postn^−/−^ and Postn^+/+^ mice (Table 1).

#### Alveolar bone histopathology

**Figure 5:**
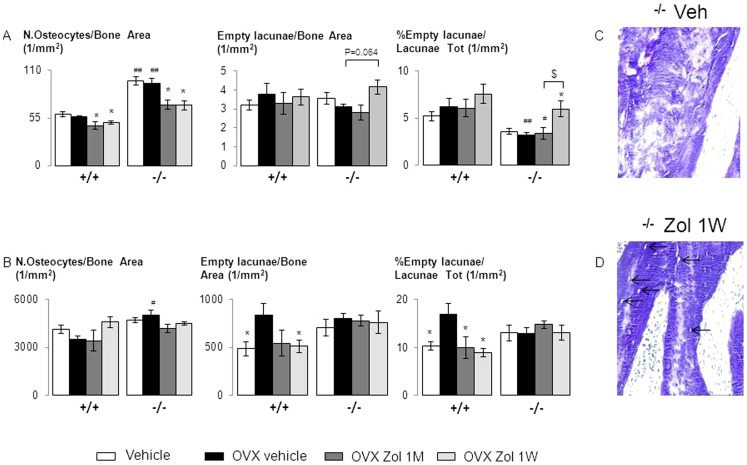
Zoledronate effect on osteocyte number and empty lacunae in jaw and femur site of *Postn^+/+^* and *Postn^−/−^* mice. Osteocyte number and empty lacunae in the jaw (A) and the mid-shaft femur (B), (C–D) Toluidine blue, (C) Full of osteocyte lacunae with osteocyte in vehicle (Veh) *Postn^−/−^*, (D) Less osteocyte and more empty lacunae (arrow) in zoledronate one injection per week (Zol 1W) vs vehicle in *Postn^−/−^* mice. * p<0.05 vs OVX vehicle in the respective genotype of *Postn^+/+^* and *Postn^−/−^*. # p<0.05, ## p<0.01 vs *Postn^+/+^* in the respective treatment. $ p<0.05 OVX Zol 1M. Bars show means (± sem).

Clinical and histological examination revealed no osteonecrotic area in the mandible of either group. Zol did not influence the size of medullar area filled by fibrous tissue (Fig. 4H). There was also no evidence of inflammatory infiltrates in any area of the mandible in relation to Zol treatment (Fig 4G). However, Zol decreased the number of osteocytes in deep bone area in both Postn^+/+^ (−17% and −11% vs veh, respectively in Zol1M and Zol1W, p<0.05) and Postn^−/−^ mice (−26% and −27% vs veh, respectively in Zol1M and Zol1W, p<0.05, Fig. 5A). In Postn^−/−^, but not Postn^+/+^ mice, Zol 1W also increased the percentage of empty lacunae (+33.9% in Zol1W vs veh, p<0.05). However, we did not observe areas of adjacent empty lacunae that define necrotic bone tissue [27] (Fig. 5C–D).

By comparison, no reduction in osteocyte number or increase in the percentage of empty lacunae with Zol was observed in long bones (Fig. 5B). On the contrary, in OVX Postn^+/+^ mice, Zol 1M and 1W significantly decreased the percentage of empty lacunae vs veh (Fig. 5B).

## Discussion

Despite the prominent benefits of bisphosphonates on preventing osteoporotic fractures and the improvement of alveolar bone and tooth loss in PD with these drugs, the occurrence of ONJ, particularly with oncological doses of i.v. zoledronate, has raised new concerns about their therapeutic margin, -i.e. the difference between the optimal effective dose and the dose at which unacceptable adverse effects occur-. Our results indicate that in absence of cancer, injury and/or severe inflammation, zoledronate at up to 4 fold the human oncological dose is safe and effective in improving jaw (and systemic) bone loss in rodents. They further indicate that suppressed bone turnover together with alterations of bone mass and quality, as observed in *Postn^−/−^* mice, is not sufficient to induce jaw bone necrosis. Periodontal disease and particularly periodontitis is characterized by inflammation of the periodontal tissues (gingival tissue, alveolar bone and periodontal ligament) caused by pathogenic microflora, loss of connective tissue and crestal alveolar bone [48]. Periostin-deficient mice fed a soft diet represent a unique genetic model of systemic and jaw bone alterations. In these conditions, crestal alveolar bone is affected as DEA increased, and the periodontal ligament appeared enlarged. Basal bone, which does not belong to the periodontal tissue, was also affected as BV/TV and fibrous areas were increased. This is sustained by an imbalance of bone remodeling secondary to a decrease of bone formation and an increase of bone resorption. However no prominent inflammatory infiltrates were observed in periodontal tissues, mucosa and bone, nor were periodontal pockets present, indicating the absence of overt PD.

In this model, even supra-pharmacological exposure to Zol, i.e 4 folds the human oncological dose, did not reactivate inflammation and/or worsen the DRI. Although, Zol decreased osteoclast and osteocyte number, and increased the percentage of empty lacunae in *Postn^−/−^*, no area of necrotic bone was observed. Hence, in absence of active inflammation, suppression of bone turnover (by BPs) together with alterations of alveolar bone mass and bone quality, i.e collagen fibers as in *Postn^−/−^* [34], is not sufficient to induce bone necrosis. Our results markedly differ from previous observations in which zoledronate was administered at various doses in animal models subjected to severe surgical procedure (ligature [27] or extraction [49], [50]), induction of PD by high sucrose diet [28], metastatic processes, bacteria inoculation [51] and/or cytotoxic drugs (dexamethasone, docetaxel…) [52]. In these models, in addition to cellular infiltration, lesions reminiscent of ONJ have sometimes been observed. Taken together these experiments suggest that injury/inflammation is an pre-requisite for the development of necrosis.

In wild-type OVX mice, Zol 1W still improved trabecular and compact bone in the femur and basal bone in the jaw, i.e similar to the oncologic dose (Zol 1M), as previously described [36]. However, in the jaw alveolar compartment, BV/TV remained lower with weekly Zol administration. The concomitant decrease of osteocytes, osteoclasts and bone remodeling (i.e low BFR) in the jaw, could suggest a lower cell viability and / or replacement rate with frequently dosed Zol, i.e low remodeling levels. In contrast, we did not observe a significant reduction of osteocyte number in the femur. Toxic effects of BPs on fibroblasts and epithelial cells have been reported in-vitro and at high concentrations [53], [54], [55], and suggested on osteocytes in vivo [56]–[57]. However despite many studies, direct effects of BPs on bone osteoblasts/osteocytes have been difficult to demonstrate in vivo using clinically relevant doses [58]. The site-specific effects of BPs on osteocytes observed in our study could be explained by some differences in the biology of jaw vs peripheral osteoblasts, which have various embryonic origins: in the long bones the osteoblast lineage derives from the trunk lateral plate mesoderm, whereas in the mandible it derives from the paraxial mesoderm [59]. It could also be explained by the different remodeling rate between these skeletal sites, which is higher in the jaw [60], potentially facilitating the accumulation of BPs in the bone matrix [61], [62]. However, after a single infusion of Zol in dogs, drug concentration in jaw and teeth was not higher than in tibia and actually lower than in pelvis or vertebrae [63]. Repeated administration of ibandronate in rats also showed the absence of preferential intake in the jaw [64].

It has been shown that the decrease of bone turnover by Zoledronate does not affect serum periostin levels or periostin immunostaining in mice [65], [66]. In contrast, in absence of periostin, high dose Zol was the least effective on the jaw BV/TV. Hence, *Postn^−/−^* mice receiving Zol 1W maintained a lower alveolar BV/TV compared to mice receiving Zol 1M, and its absolute value did not differ from placebo. Moreover, in these mice, Zol 1W increased the number of empty osteocytic lacunae. Basal bone turnover is lower in *Postn^−/−^*, however the decrease of bone formation indices in response to Zol was not more pronounced in absence of periostin. Hence, it is unlikely that the poor gain of alveolar BV/TV under Zol in *Postn^−/−^* mice could be explained by an over-suppression of bone turnover.

In a model of fibrosis [67], interleukin-4, -13, two anti-inflammatory cytokines, induced periostin expression, whereas during periodontal inflammation periostin staining is down-regulated [68]. On another side, *Postn^−/−^* mice have latent PD, and inflammatory cells produce cytokines that decrease osteoblast functions. However, *Postn^−/−^* mice did not present an increased infiltration of jaw bone by inflammatory cells in response to Zol. We previously reported that loading and PTH increases periostin expression in osteocytes [34], and that periostin decreases the expression of apoptotic molecules such as caspase 3 [47], potentially through direct binding to intergrin αVβ5 which activate downstream FAK and Akt pathways. In others tissues, increased periostin expression have also been suggested to represent an adaptative cell response to maintain cell survivals against an environmental stress [69], [70], [71]. Therefore, it is possible that absence of periostin promoted BPs pro-apoptotic effects on osteocytes [61]–[62], which together with low bone turnover would explain the persistence and accumulation of empty osteocytic lacunae. We cannot exclude that with a higher number of animals and a longer duration of treatment the accumulation of empty osteocytic lacunae would have led to the detection of bone necrosis.

To conclude, zoledronate improved systemic and alveolar bone alterations in OVX *Postn^−/−^* mice, without producing jaw bone necrosis.
